# Revisit to diffraction anomalous fine structure

**DOI:** 10.1107/S1600577514015148

**Published:** 2014-10-02

**Authors:** T. Kawaguchi, K. Fukuda, K. Tokuda, K. Shimada, T. Ichitsubo, M. Oishi, J. Mizuki, E. Matsubara

**Affiliations:** a Department of Materials Science and Engineering, Kyoto University, Kyoto 606-8501, Japan; b Office of Society-Academia Collaboration for Innovation, Kyoto University, Kyoto 611-0011, Japan; c Graduate School of Engineering, Kyoto University, Kyoto 606-8501, Japan; d School of Science and Technology, Kwansei Gakuin University, Hyogo 669-1337, Japan

**Keywords:** diffraction anomalous fine structure, powder diffraction, site specification, logarithmic dispersion relation, non-iterative method

## Abstract

The diffraction anomalous fine structure (DAFS) method that is a spectroscopic analysis combined with resonant X-ray diffraction enables the determination of the valence state and local structure of a selected element at a specific crystalline site and/or phase. This method has been improved by using a polycrystalline sample, channel-cut monochromator optics with an undulator synchrotron radiation source, an area detector and direct determination of resonant terms with a logarithmic dispersion relation. This study makes the DAFS method more convenient and saves a large amount of measurement time in comparison with the conventional DAFS method with a single crystal. The improved DAFS method has been applied to some model samples, Ni foil and Fe_3_O_4_ powder, to demonstrate the validity of the measurement and the analysis of the present DAFS method.

## Abstract 2

The diffraction anomalous fine structure method has been revisited by applying this measurement technique to polycrystalline samples and using an analytical method with the logarithmic dispersion relation.

## 1. Introduction   

The diffraction anomalous fine structure (DAFS) method is a spectroscopic analysis method established by coupling X-ray diffraction (XRD) and X-ray absorption fine structure (XAFS) (Stragier *et al.*, 1992 ▶; Tweet *et al.*, 1992 ▶; Pickering *et al.*, 1993 ▶). In the DAFS method, an XAFS-like spectrum is determined from the energy dependence of structure factors in XRD around the absorption edge of a selected element. Thus, the local atomic configuration and chemical state can be determined around a certain element at a particular crystallographic site of a unit cell and also in a particular crystalline phase of a sample. Pioneering DAFS studies, including theoretical introductions, have been reported (Renevier *et al.*, 1997 ▶; Cross *et al.*, 1998 ▶; Meyer *et al.*, 1999 ▶; Proietti *et al.*, 1999 ▶; Lee *et al.*, 2006 ▶; Joly *et al.*, 2008 ▶) and reviewed (Sorensen *et al.*, 1994 ▶; Favre-Nicolin *et al.*, 2012 ▶; Palancher *et al.*, 2012 ▶; Zschornak *et al.*, 2014 ▶). However, there have been few reports on the application of DAFS to practical materials, which often require specific types of investigations (such as a time-series variation associated with the durability and degradation of product performances), presumably because of the two scientific challenges discussed below.

First, DAFS measurements are time-consuming. In the early DAFS measurements, a single crystal was generally used to attain a high signal-to-noise ratio (Stragier *et al.*, 1992 ▶; Renevier *et al.*, 2003 ▶). The acquisition time for an entire spectrum was quite long because sample alignment is needed at each incident energy around the absorption edge to satisfy the diffraction condition. However, this alignment is not required for a polycrystalline sample, although the lower scattering power and intense fluorescence of the sample may hinder the accurate collection of the DAFS spectrum (Pickering *et al.*, 1993 ▶; Palancher *et al.*, 2012 ▶). Recent steady progress in synchrotron science has encouraged us to develop a more efficient way to collect a quality DAFS spectrum in a reasonable amount of time.

Second, a common analytical method of DAFS spectra by iterative fitting is mathematically challenging. In this analysis, the real and imaginary parts of resonant dispersion terms, 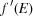 and 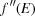, must be extracted from resonant XRD intensities. Stragier *et al.* (1992 ▶) divided a DAFS spectrum into smooth and oscillating parts and extracted spectrum oscillations by fitting the smooth part with a theoretically calculated profile. This calculation method has subsequently inspired analyses of data for extended diffraction anomalous fine structure (EDAFS), which is analogous to extended X-ray absorption fine structure (EXAFS) (Favre-Nicolin *et al.*, 2012 ▶), but the method is not applicable to analyses of data for diffraction anomalous near-edge structure (DANES), which corresponds to X-ray absorption near-edge structure. Pickering *et al.* (1993 ▶) and Vacínová *et al.* (1995 ▶) proposed the iterative-fitting method, by which an entire DAFS spectrum is fitted using presumed 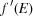 or 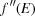 values. Their method allows discussion of valence states as well as local structures around a resonant atom at a particular site and/or phase from both DANES and EDAFS. However, the inherent ambiguity of DAFS analyses, which arises because the number of fitting parameters including an entire spectrum exceeds the number of experimental data points from one spectrum, remains a problem. Namely, no unique solutions are obtained without mathematical constraint. A direct extraction of the resonant dispersion terms should reduce the number of measured spectra and circumvent the fitting ambiguity, eventually reducing the measurement time.

In this study, we revisit the DAFS method by adopting advanced synchrotron X-ray optics and an area detector, both of which have advanced significantly since the 1990s, when DAFS was first demonstrated, and by proposing a non-iterative fitting method of DAFS spectra based on a ‘logarithmic dispersion relation’ (denoted LDR hereafter) (Burge *et al.*, 1976 ▶) that has been utilized for the direct phase determination of infrared (Fahrenfort, 1961 ▶) and X-ray/neutron (Clinton, 1993 ▶) reflectivity spectra. We first describe an analytical method for DAFS with LDR and experimental set-ups. We then demonstrate and highlight the validity of our improved DAFS method with two reference materials.

## 2. Analysis of DAFS spectra   

The total diffracted power of a powder sample from reflection *hkl* is given by
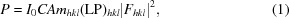
where 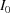 is the incident beam intensity, *C* is a constant independent of the incident photon energy *E* and *hkl*, and *A* is the absorption factor (Warren, 1990 ▶). The terms 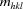, 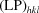 and 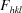 are the multiplicity factor, Lorentz–polarization factor and structure factor of *hkl*, respectively. The structure factor is given by
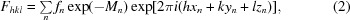
where 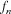 is the atomic scattering factor of the *n*th atom in the unit cell with temperature factor 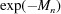. The summation over *n* involves the positions 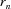 of the different atoms in the unit cell, with 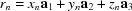 in terms of the fractional coordinates 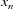, 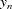 and 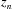 along the lattice vectors 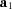, 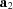 and 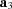, respectively. The atomic scattering factor is represented as a complex number, 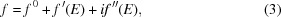
where 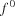 is the atomic form factor and 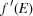 and 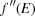 are the real and imaginary corrections for dispersion, respectively. Note that 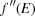 can be directly compared and processed as an XAFS-like spectrum because 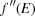 is proportional to 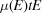 (Pickering *et al.*, 1993 ▶), where 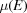 and *t* are the linear absorption coefficient and thickness of a sample, respectively. Additionally, 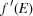 is related to 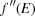 by their mutual Kramers–Kronig relationships. Thus, a goal of DAFS analysis is to extract an 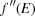 [or 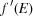] spectrum from an observed energy spectrum of diffraction intensity, *i.e.* a DAFS spectrum, 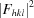.

The structure factor is also defined as the product of the absolute value of 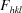 as the amplitude and the phase term,
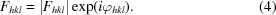
The LDR is the Kramers–Kronig relation between 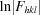 and 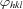. The phase 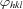 can be obtained when the spectrum of 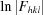 is known over a wide energy range around the absorption edge, and the 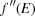 spectrum can be determined from this obtained phase and the DAFS spectrum. In computation of the phases with LDR, we must take into consideration various corrections due to the inevitable limitation of the experimental data available. Experimentally, we can diminish the error by measuring the energy region far below the absorption edge as well as at high energy where the oscillations vanish (Roessler, 1965 ▶). The value of 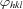 calculated from LDR without any data correction has an offset. In the present study, we added correction terms to the LDR equation (Roessler, 1965 ▶, 1966 ▶) to cancel the offset.

From LDR, the phase 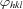 in equation (4) is given by (Roessler, 1965 ▶, 1966 ▶; Burge *et al.*, 1976 ▶)
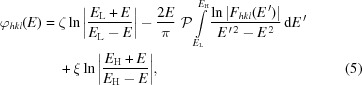
where 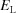 and 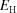 are the low- and high-energy limits of DAFS measurements and 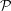 denotes the Cauchy principal value of the integral. The first term in the right-hand side of equation (5) corresponds to the entire contribution of the energy region below 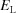, the third to the contribution from all energies above 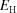, and the second is the contribution from the region with experimental data. In the present study, the unknown quantities 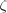 and 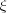 were uniquely determined by realizing that the phase shift is zero at energies below the absorption edge in centrosymmetric systems. In noncentrosymmetric systems, in which we newly define the average phase 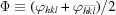, the phase calculated in equation (5) by using 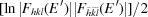 instead of 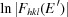 is approximated by zero at energies below the absorption edge. In addition, ζ and ξ for Φ of *hkl* and 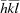 can be approximated by these expressions for ϕ, respectively, because the DAFS spectra of *hkl* and 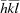 reflections are coincident far from the absorption edge. Thus, ζ and ξ in equation (5) are determined by computing Φ and its correction terms in noncentrosymmetric systems.

By combining and arranging the real and imaginary parts of equations (2)–(4) [Disp-formula fd4], we can derive the following relations,
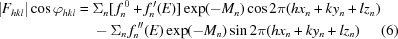
and
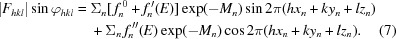
The left-hand sides of (6) and (7) are experimentally determined for an observed *hkl* reflection. Measuring DAFS spectra of *hkl* and 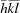 reflections separately yields an XAFS-like spectrum of a certain phase, which is the second term of the right-hand side in (7). Based on the crystal structure, we separate 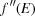 at each site as follows. Given the number of unknown 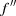, we should solve the same number of sets of equations (6) and (7) obtained from an adequate number of different DAFS spectra. When we use polycrystalline samples, this method can only be applied to a centrosymmetric crystalline structure in which the sine terms in (6) and (7) do not appear due to symmetry. This restriction is not the case in single-crystal measurements. Even in a noncentrosymmetric single crystal, the simultaneous equations can be solved, as both *hkl* and 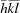 reflections with different DAFS spectra (Meyer *et al.*, 1998 ▶) can be separately measured.

## 3. Experimental   

A polycrystalline Ni foil and an Fe_3_O_4_ powder were prepared for the samples. The Ni foil for an XAFS standard sample of 6 µm in thickness was used to allow direct comparison of 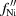 determined from DAFS with its XAFS spectrum. Fe_3_O_4_ powder (4.1 mg) was pellet­ized into a 10 mm-diameter disk with 35.3 mg BN powder as a diluent, corresponding to 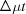 of approximately 1.3 at the absorption edge. The samples were mounted on a Huber eight-axis goniometer in asymmetric transmission geometry.

**Figure 1:**
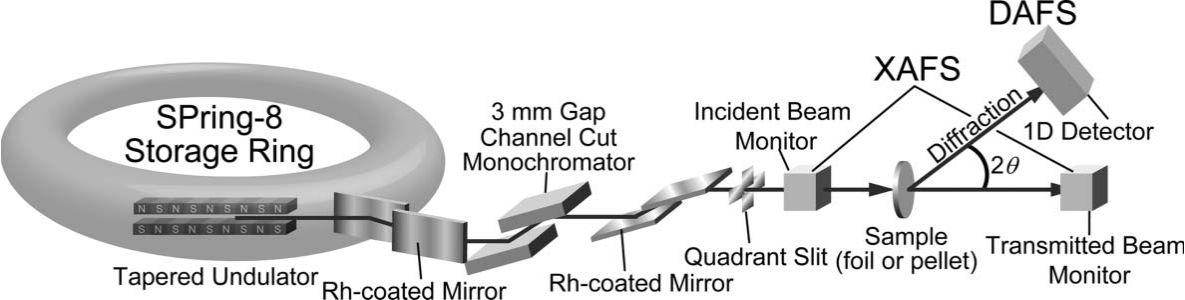
A schematic of the optical geometry used in this study on beamline BL28XU at SPring-8 in Japan.

DAFS measurements were carried out at beamline BL28XU (Tanida *et al.*, 2014 ▶), SPring-8, Japan. Fig. 1 ▶ shows the optical arrangement and experimental set-up used in this study. A distinct feature of the optics was a Si (111) channel-cut monochromator with a 3 mm gap in combination with two pairs of horizontal and vertical Rh-coated mirrors upstream and downstream of the monochromator for elimination of high-order harmonics. Due to the small gap of the monochromator, the vertical shift of the beam during energy scanning for collection of a DAFS spectrum was limited to within 20 µm, which is much smaller than the total vertical width of the incident beam. Thus, by shaping the incident beam of 500 µm horizontal and 200 µm vertical widths with a quadrant slit, the position and approach angle of the incident beam were maintained at the sample position. Ion chambers with He gas flow were placed on both sides of the sample to monitor intensities of the incident and transmitted beams for the XAFS measurement and the absorption correction of the DAFS spectra. A one-dimensional detector, Mythen (Dectris; Baden, Switzerland), was mounted on the 2θ axis and used to measure diffraction profiles. The distance from the sample to the detector was 1048 mm, and the detective area and width of each pixel were 64 mm × 8 mm and 50 µm, respectively, which ensured simultaneous observations of 3.5° with 0.0027° resolution in 2θ. Evacuated flight paths were placed between the incident ion chamber and the sample and also between the sample and the detector to reduce attenuation and scattering of X-rays by air.

The DAFS spectrum of the Ni foil was measured using 111 diffraction peaks at 155 energy points from 8.041 to 8.431 keV. The accumulation time of each diffraction peak was approximately 2 s. Two DAFS spectra of Fe_3_O_4_ were obtained from 220 and 311 diffractions at 451 energy points between 6.820 and 8.290 keV. The accumulation time was approximately 10 s for 220 and 1 s for 311. The observed peak profile at each energy was fitted by the least-squares method using a linear background with the split pseudo-Voigt peak function described by Toraya (1990 ▶). The integrated peak intensities in equation (1) were corrected for the incident X-ray intensity, the Lorentz–polarization factor and the absorption factor calculated from the experimental absorption coefficients from the XAFS spectrum and shape of the sample. The scaling factor for conversion to the electron unit was determined by comparing the observed integrated intensities at the lowest and highest energies in the experimental DAFS spectra with those calculated from the structural parameters refined by the Rietveld method (Izumi & Momma, 2007 ▶), with theoretical resonant terms calculated by the Cromer–Lieberman method (Sasaki, 1989 ▶).

## 4. Results and discussion   

**Figure 2:**
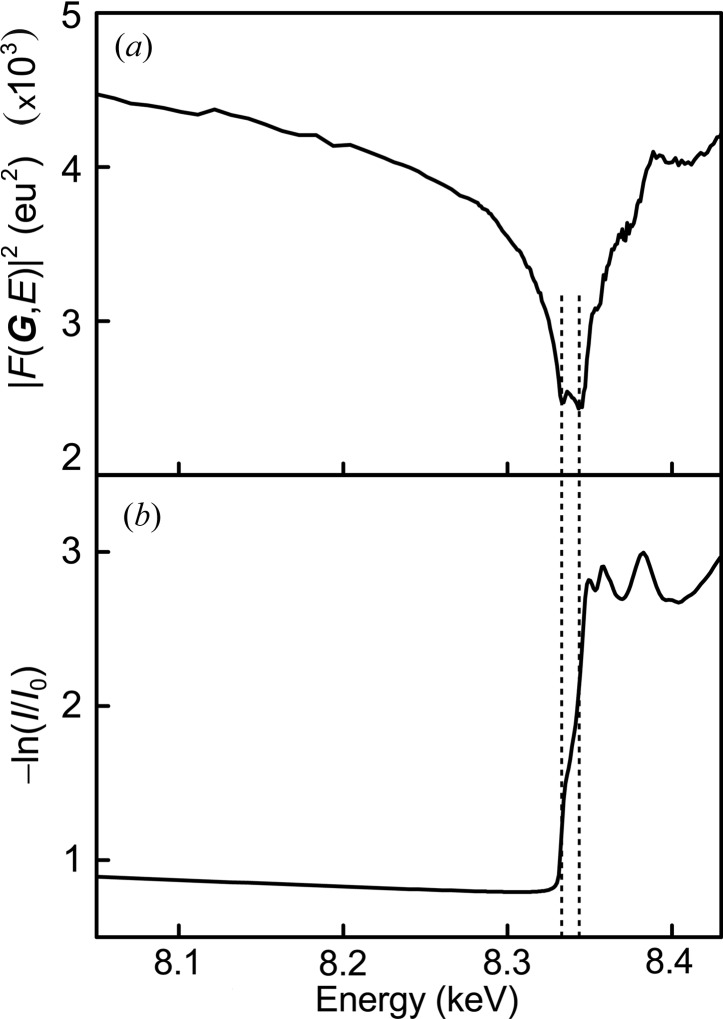
(*a*) DAFS spectrum obtained from Ni 111 diffraction and (*b*) XAFS spectrum simultaneously measured.

In Fig. 2 ▶, the DAFS spectrum of Ni 111 obtained from the polycrystalline Ni foil is compared with the Ni XAFS spectrum. The total measurement time is approximately 6 min, which is comparable with that of the simplest XAFS measurement in transmission mode. The DAFS spectrum monotonically decreases toward the Ni *K*-edge at approximately 8.340 keV owing to the resonant effect in 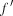. The spectrum rapidly increases with oscillations above the absorption edge. The two local minima around the cusp at 8.333 and 8.343 keV in the DAFS spectrum correspond to the two inflection points of the XAFS spectrum at the absorption edge, indicating that the Kramers–Kronig relation between absorption and scattering is similar to a differentiation (Sorensen *et al.*, 1994 ▶). We limit the following discussion to the DANES region with significant changes in XRD intensities to confirm the validity of the present DAFS method. In the EDAFS region, the oscillation of the spectrum tends to be disturbed by random noise because of tiny changes in the XRD intensities.

**Figure 3:**
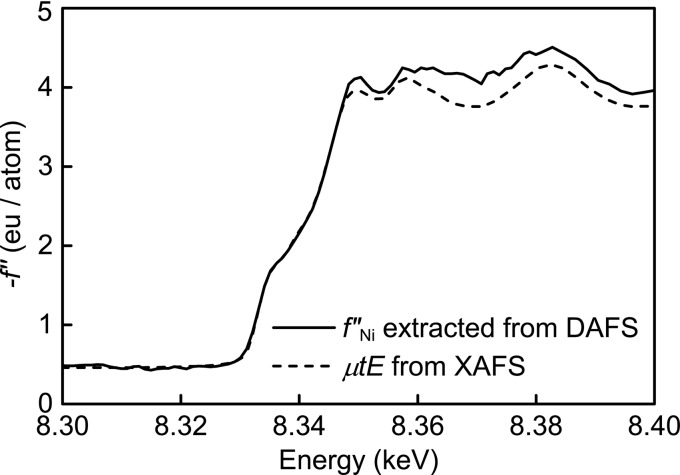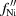 extracted from a 111 DAFS spectrum by LDR (solid line) and μ*tE* obtained from an XAFS measurement (broken line). The values of 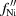 have some offset and μ*tE* is scaled by theoretical values (Sasaki, 1989 ▶) for comparison.

The solid line in Fig. 3 ▶ shows 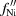 extracted from the Ni 111 DAFS spectrum by
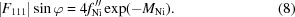
The XAFS spectrum (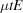) in the broken line must be equivalent to 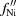 because all Ni atoms occupy only one symmetrically equivalent site in the face-centered cubic lattice. The XAFS spectrum is rescaled with the theoretical value reported in the literature (Sasaki, 1989 ▶). Notably, the spectrum of 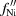 shows good agreement with that of 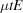 in terms of edge shape and oscillation, which clearly indicates the validity of the present DAFS measurement and analysis.

**Figure 4:**
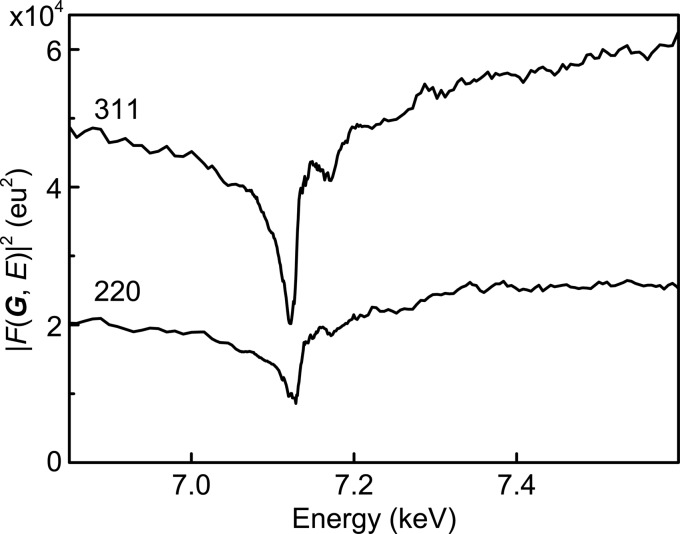
DAFS spectra of Fe_3_O_4_ from 311 and 220 diffraction.

**Figure 5:**
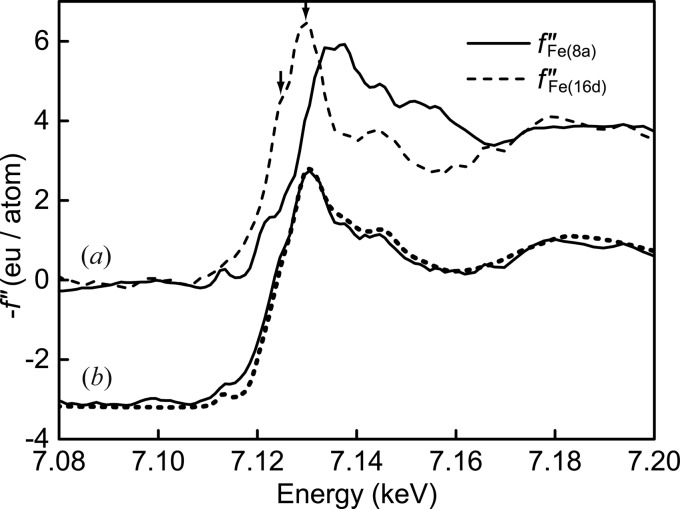
(*a*) Fe at the 8*a* tetrahedral site (solid line) and 16*d* octahedral site (broken line) extracted from DAFS spectra. (*b*) Averaged spectrum at each site from the DAFS method (solid) and μ*tE* obtained from XAFS (dotted line) with an offset of −3.

Next, to demonstrate site-selective analysis on the basis of our DAFS method, we investigated a powder sample of inverse spinel Fe_3_O_4_, in which tetrahedral and octahedral sites are occupied by Fe^3+^ and a mixture of Fe^2+^/Fe^3+^ (Lorimier *et al.*, 2003 ▶), respectively. The DAFS spectra obtained for Fe_3_O_4_ 220 and 311 are shown in Fig. 4 ▶. As with the Ni foil, the local minima in the DAFS spectra of Fe_3_O_4_ corresponding to the absorption edge, and the oscillations above the edge, can be seen. Unlike the Ni foil case, the spectra of 220 and 311 are markedly different, reflecting the different contributions from the tetrahedral and octahedral sites. The imaginary parts of the resonant dispersion terms of Fe at tetrahedral (8*a*) and octahedral (16*d*) sites in Fe_3_O_4_ were calculated by solving the simultaneous equations derived from equation (7),
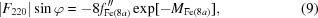


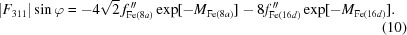
In the above derivation, we used the atomic coordinates of ‘*origin choice 2*’ in *International Tables for Crystallography* (Vol. A; Hahn, 2006 ▶) with centrosymmetry in space group 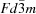. Note that ‘*origin choice 1*’ is also available by taking account of the phase shift below the absorption edge. The spectra of 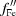 at the 8*a* and 16*d* sites are compared in Fig. 5 ▶(*a*). A distinctive pre-edge peak is observed at 7.114 keV in 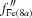 for the tetrahedral site, while the pre-edge peak of 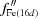 for the octahedral site is broad and weak. The pre-edge peak at 8*a* appears because of the transition from the Fe 1*s* orbital to the *p* component of the *p*–*d* hybrid orbital (Frenkel *et al.*, 1999 ▶). In contrast, at 16*d* sites, the allowed transition weakly occurs due to the absence of the hybridization, and only a weak peak arising from the quadrupole transition of Fe 1*s* to Fe 3*d* is observed. In the 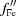 spectrum at 16*d*, there are two shoulders at the main edge, indicated by arrows, due to the contributions from Fe^2+^ and Fe^3+^ (Sasaki, 1995 ▶; Kobayashi *et al.*, 1998 ▶). These distinctive profiles are consistent with previous DAFS works (Kobayashi *et al.*, 1998 ▶; Frenkel *et al.*, 1999 ▶; Joly *et al.*, 2008 ▶). By weighting the experimental 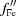 spectrum at each site [see Fig. 5 ▶(*a*)] in response to the number of atoms at the site, an averaged 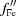 spectrum comparable with 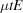 from the XAFS spectrum was computed and is shown as a solid line in Fig. 5 ▶(*b*). The agreement of the two spectra demonstrates the strong potential of site-selective XAFS in our improved DAFS method and re-emphasizes the advantages of the DAFS method.

## 5. Conclusion   

In the present study, we revisited the DAFS method by applying this measurement technique to polycrystalline samples and using an analytical method with LDR. The validity of this improved DAFS method was demonstrated through analyses of centrosymmetric polycrystalline samples. Using polycrystalline samples in the DAFS method is advantageous in terms of total experimental time. In addition, the combination of optics for an undulator synchrotron radiation source and an area detector enables us to collect a DAFS spectrum in a reasonable amount of measurement time without losing data quality from the polycrystalline sample, which may promote its application to polycrystalline materials widely used at present in industry. The analytical method using LDR proposed in this study reduces the number of measured spectra necessary for DAFS analyses and yields a direct solution of the resonant terms readily without the inherent ambiguity of the conventional iterative-fitting method. Such a quick and reliable DAFS method, which is both site and spatial selective, will be a breakthrough tool for understanding the structures and chemical states of complex systems, including multiphase or multielement crystalline samples.
